# The impact of consent on observational research: a comparison of outcomes from consenters and non consenters to an observational study

**DOI:** 10.1186/1471-2288-8-15

**Published:** 2008-04-03

**Authors:** Una Macleod, Graham CM Watt

**Affiliations:** 1 General Practice and Primary Care, Division of Community Based Sciences, Faculty of Medicine, University of Glasgow, 1 Horselethill Road, Glasgow, G12 9LX, UK

## Abstract

**Background:**

Public health benefits from research often rely on the use of data from personal medical records. When neither patient consent nor anonymisation is possible, the case for accessing such records for research purposes depends on an assessment of the probabilities of public benefit and individual harm.

**Methods:**

In the late 1990s, we carried out an observational study which compared the care given to affluent and deprived women with breast cancer. Patient consent was not required at that time for review of medical records, but was obtained later in the process prior to participation in the questionnaire study. We have re-analysed our original results to compare the whole sample with those who later provided consent.

**Results:**

Two important findings emerged from the re-analysis of our data which if presented initially would have resulted in insufficient and inaccurate reporting. Firstly, the reduced dataset contains no information about women presenting with locally advanced or metastatic cancer and we would have been unable to demonstrate one of our initial key findings: namely a larger number of such women in the deprived group. Secondly, our re-analysis of the consented women shows that significantly more women from deprived areas (51 v 31%, p = 0.018) received radiotherapy compared to women from more affluent areas. Previously published data from the entire sample demonstrated no difference in radiotherapy treatment between the affluent and deprived groups.

**Conclusion:**

The risk benefit assessment made regarding the use of medical records without consent should include the benefits of obtaining research evidence based on 100% of the population and the possibility of inappropriate or insufficient findings if research is confined to consented populations.

## Background

Many research studies which have led to improvements in public health benefits have relied on the use of data from personal medical records [1]. In particular, retrospective review of medical records has been carried out for many years in order to answer questions related to the coverage and equity of health care. When neither patient consent nor anonymisation is possible, the case for accessing such records for research purposes depends on an assessment of the probabilities of public benefit and individual harm. The past decade has seen a shift in attitude towards using data derived from medical records without patient consent. Previously, ethics approval for studies involving retrospective use of data was based on a range of considerations including the relevance and usefulness of the research question, scientific peer review, suitable data collection methods and data security. Subject to these caveats, many studies have been carried out, which included almost 100% coverage of the target population.

In the current ethical and research governance climate, primacy tends to be given to considerations of individual autonomy [2] over considerations of public benefit. It has become a common view that explicit consent is required in order to use identifiable personal data for research [3]. However, a growing body of evidence demonstrates that studies relying solely on patient consent may produce findings that are unrepresentative [4-7] or misleading [8]. In some instances, ethics committees have permitted an opt-out arrangement where researchers have been required to first contact the patient for permission to review records, and asking them to reply if they object to their records being reviewed; this is not yet in universal practice, particularly in cases where, due to the requirements of the particular study, records cannot be anonymised. These arguments have not however been applied to audit which is considered an essential part of good clinical practice, and for which patient consent is not required.

Recent analysis of changes in relevant legislation brought about by the Data Protection Act and the Humans Right Act, suggest that these laws need not prevent researchers from using identifiable data without consent, provided there is a clear public benefit [9]. In this paper, we provide further evidence of how limiting the use of identifiable patient data may act against the public interest.

## Methods

In the late 1990s, we compared the care provided by the NHS in Glasgow for all 421 women with breast cancer living in affluent and deprived areas [10-12]. Population coverage by the study was 100% based on ascertainment of cases from cancer registration and 99% based on hospital case notes. We then opted to concentrate on patients who had operable breast cancer (n = 366) and obtained general practice records relating to 76% (n = 278) of these women. A postal questionnaire was sent to women whom we knew to be alive at the time of the general practice records data (n = 218) collection and whose general practitioners confirmed they were alive and well just prior to posting the questionnaire. Cases were "lost" for the purpose of this retrospective study at two points: at the general practice data collection stage and at the questionnaire stage. The issue at the point of reviewing general practice records was that we required practices to facilitate us collecting data from their records; a number of practices did not engage with the study with respect to this after many contacts by letter, fax and phone and so we were only able to review 78% of potential records: we did not request practices to obtain consent from patients. As we were contacting women several years after their diagnosis of cancer, we decided to check with general practitioners before sending out questionnaires as to whether the women were alive and well.

Ethical approval was obtained for the study, which combined comprehensive record review with a patient questionnaire five years after diagnosis. Patient consent was not required at that time for the review of records, but was given later in the process when women were contacted and invited to participate in the questionnaire study [12]. In this paper, we summarise the original findings, and add a parallel set of findings, based only on review of the medical records of the 177 women who took part in the questionnaire survey, and assuming that, if they had been asked, these women would also have consented to the review of their medical records. For the purpose of clarity, these women are referred to here as the 'consented' sample.

## Results

In our original study, the virtually complete sample of hospital records allowed us to show equitable provision of the main treatments for breast cancer – surgery, radiotherapy and chemotherapy – according to tumour stage. We also showed that pathological staging information was not available for 12% of women, mostly from deprived areas, who presented with more locally advanced or metastatic tumours [10,11].

In the new analyses reported here, restricted to the hospital records of the women who gave consent by returning questionnaires, two findings emerged which are different from those we reported earlier.

First, although the reduced dataset shows similar findings to the main study (with wider confidence limits) with respect to treatments by tumour staging in women presenting with early tumours, it contains no information about the 12% of women presenting with locally advanced or metastatic cancer (Table 1).

**Table 1:** Pathological prognostic factors and clinical stage at presentation for women with breast cancer living in affluent and deprived areas (whole sample and questionnaire respondents)

	**AFFLUENT n (%)**		**DEPRIVED n (%)**		**Chi squared test result**	
**PATHOLOGICAL PROGNOSTIC FACTORS**						

SIZE*	***WHOLE SAMPLE*** ***n = 136***	***CONSENTED SAMPLE*** ***n = 72***	***WHOLE SAMPLE*** ***n = 194***	***CONSENTED SAMPLE*** ***n = 91***	***WHOLE SAMPLE***	***CONSENTED SAMPLE***

**0 – 19 mm**	70 (51.5%)	**42 (58.3%)**	106 (54.6%)	**54 (59.3%)**	**X^2 ^= 0.53**	**X^2 ^= 0.04**
**20 – 49 mm**	62 (45.6%)	**29 (40.3%)**	81 (41.8%)	**36 (39.6%)**	**DF = 2**	**DF = 2**
**>50 mm**	4 (2.9%)	**1 (1.4%)**	7 (3.6%)	**1 (1.1%)**	**p = 0.76**	**p = 0.98**
						
GRADE*	*n = 110*	***n = 59***	*n = 156*	***n = 72***		
**1**	17 (15.5%)	**9 (15.3%)**	30 (19.2%)	**18 (25.0%)**	**X^2 ^= 0.66**	**X^2 ^= 1.83**
**2**	67 (60.9%)	**39 (66.1%)**	92 (59.0%)	**42 (58.3%)**	**DF = 2**	**DF = 2**
**3**	26 (23.6%)	**11 (18.6%)**	34 (21.8%)	**12 (16.7%)**	**p = 0.72**	**p = 0.34**
						
NODAL STATUS*	*n = 128*	***n = 72***	*n = 196*	***n = 93***		
**Positive**	48 (37.5%)	**24 (33.3%)**	72 (36.7%)	**28 (30.1%)**	**X^2 ^= 0.01**	**X^2 ^= 0.196**
**Negative**	80 (62.5%)	**48 (66.6%)**	124 (63.3%)	**65 (69.9%)**	**DF = 1** **p = 0.88**	**DF = 2** **p = 0.736**
						
**CLINICAL STAGE AT PRESENTATION**						
						
	*n = 156*		*n = 260*			
**Early**	146 (93.6%)	**100%**	220 (84.6%)	**100%**	**X^2 ^= 7.42**	**N/A**
**Locally advanced or metastatic**	10 (6.4%)		40 (15.4%)		**DF = 1** **p = 0.006**	

* Size, grade and nodal status only potentially available for the 366 women (146 affluent and 220 deprived) who had operable breast cancer.

*Total n for whole sample < 366 due to missing data

**Total n for consented sample < 177 due to missing data

The second different finding shows that significantly more women from deprived areas (51 v 31%, p = 0.018) received radiotherapy compared to women from more affluent areas (Table 2). Other findings, although with a smaller sample, were not significantly different from those originally found and published.

**Table 2:** Surgical treatment, radiotherapy and adjuvant therapy for women living in affluent and deprived areas (whole sample and questionnaire respondents)

	**AFFLUENT n (%)**		**DEPRIVED n (%)**		**Chi squared test result**	
	*WHOLE SAMPLE*	***CONSENTED SAMPLE***	*WHOLE SAMPLE*	***CONSENTED SAMPLE***	*WHOLE SAMPLE*	***CONSENTED SAMPLE***

**BREAST SURGERY**	n = 142*	n = 75**	n = 215*	n = 97**		
Mastectomy	64 (45.1%)	**37 (49.3%)**	104 (48.4%)	**43 (44.3%)**	X^2 ^= 0.37 DF = 3	**X^2 ^= 0.43 DF = 1**
Conservation	78 (54.9%)	**38 (50.7%)**	111 (51.6%)	**54 (55.7%)**	p = 0.54	**p = 0.54**
**AXILLA SURGERY**	n = 129*	n = 70*	n = 196 *	n = 94**		
Clearance	123 (95.3%)	**68 (97.1%)**	146 (74.5%)	**69 (73.4%)**	X^2 ^= 23.73 DF = 1	**X^2 ^= 16.4 DF = 1**
Sampling	6 (4.7%)	**2 (2.9%)**	50 (25.5%)	**25 (36.6%)**	p = 0.0000	**p = 0.000**
**RADIOTHERAPY**	*n = 146* 54 (37.0%)	n = 72** 22 (30.6%)	*n = 220* 90 (40.9%)	n = 98** 50 (51.0%)	X^2 ^= 0.56 DF = 1 p = 0.45	**X^2 ^= 5.82 DF = 1 p = 0.018**
**CHEMOTHERAPY**	*n = 146* 29 (19.9%)	n = 75** 13 (16.9%)	*n = 220* 30 (13.6%)	n = 100** 15 (15%)	X^2 ^= 2.51 DF = 1 p = 0.11	**X^2 ^= 0.116 DF = 1 p = 0.84**
**ENDOCRINE THERAPY**	*n = 146* 128 (87.7%)	***n = 77*** **71 (92.2%)**	*n = 220* 196 (89.1%)	***n = 100*** **92 (92.0%)**	X^2 ^= 0.17 DF = 1 p = 0.67	**X^2 ^= 0.003 DF = 1 p = 1.0**

*Total n for whole sample < 366 due to missing data

**Total n for consented sample < 177 due to missing data

Although the response rate to the questionnaire was 81%, the women returning a questionnaire comprised only 48% of the original population of women with early breast cancer. Missing categories comprised women who had died (n = 20) or left the area (n = 3), and practices which declined to take part in study (n = 19), did not respond to requests to see records after numerous contacts (n = 46), or did not confirm that the patient was alive and well at time of survey (n = 60).

## Discussion

### Main findings of this study

In re-analysing the data from our original study, we have demonstrated different results based on the virtually complete original patient sample and the reduced consented sample. These differences illustrate some of the consequences and challenges of confining research using personal medical records to patients who have given explicit consent.

The original analyses, based on records review without patient consent, provided largely reassuring information for the NHS, concerning the equity of provision of major treatments for women with breast cancer, while avoiding the uncertain, incomplete and potentially spurious nature of results based on partial data. The information regarding clinical staging which we obtained from medical records, and which had been missed in a previous study based on pathological records [13], is an important research finding and has contributed to the debate regarding the reasons for the poorer survival of women form socio-economically deprived areas with breast cancer.

The purpose of this part of the original study was to ensure comparability with previous work by Carnon et al [13]. They studied the relationship between socio-economic deprivation and pathological prognostic factors in women with breast cancer, in an attempt to explain the known poorer survival in socio-economically deprived areas. They reported that this survival difference was not related to the stage of disease at the time of presentation. We confirmed their findings [10] and also produced the new information that more women from deprived areas compared to affluent areas presented with locally advanced or metastatic tumours. This is a small, but important group of patients, whose exclusion from case series can produce misleading results. The earlier study [13] had been a study of pathological records and so had not included those patients who did not proceed to surgery and therefore for whom prognostic pathological factors were not available. Although many of the woman with advanced or metastatic cancer would have been deceased by the time of our data collection and so we would likely have been able to obtain their data without consent, we would argue that having the benefit of the whole sample enabled us to produce these comparisons with some confidence.

With respect to our second finding reported here, it is likely, had we published the spurious finding, based on smaller numbers, regarding access to radiotherapy that it 0 could have prompted unnecessary concern, and further research relating to how well women were informed about treatment options, and also about potential inappropriate exposure to radiotherapy.

### Limitations of this study

This study presents a hypothetical worst-case scenario based on a number of assumptions. Our initial assumption is that our study is less likely to receive ethical approval in the current climate. However, some ethics committees may be content to provide ethical approval for a retrospective study of this nature carried out by a suitably qualified researcher within an appropriate context. In addition, the missing data from Table 1 may have been available from death records, to which such stringent conditions may not be applied. We have also assumed that the women who completed questionnaires are likely to have consented to review of their medical records. This assumption is likely to have under-estimated the level of consent as women who did not complete the questionnaire may still have been willing for their records to be reviewed.

A further limitation is that before we were able to ask patients to take part in the study by completing the questionnaire, there was a degree of 'gate-keeping' by general practitioners who could either not engage with the study or decided that their patient wasn't suitable to be included. We were unable to determine which of these explanations was most pertinent in this study. Gate keeping by clinicians is a feature of studies from health service lists, and may have both positive and negative aspects from a research point of view. On the one hand, clinicians often screen potential lists of participants to exclude approaches in cases of severe illness or other circumstances where an approach would be inappropriate; on the other hand, clinicians may have no interest in facilitating research and relegate this task below competing demands on their time. In either situation, the net effect in a study such as ours is to increase loss to follow-up. The role that clinicians may have in gate-keeping in this way is an important and under-researched area.

### Comparison with other studies

This study adds to a growing body of evidence regarding the implications of confining research to data for which expressed and specific consent has been given. Others have shown that consented populations are likely to be unrepresentative by age or gender [5-7] or may have different clinical outcomes [4,8]. We have added to this by demonstrating the potential for erroneous conclusions.

In retrospective studies, the proportion of patients who give informed consent for research access to their records is affected mainly, not by their individual responses, but by the numbers whom it is possible to contact. Obtaining research evidence based on 100% of the population may therefore be impossible without the use of identifiable data from medical records. Surveys have shown that most of the public considers the use of identifiable data by cancer registries acceptable [14]. However others have shown that there is some public concern about the use of data without consent and demonstrated the need for further research in this area [15].

### Implications of this study

Analyses confined to the hospital records of women who consented to the postal questionnaire survey, showed a spurious finding concerning the provision of radiotherapy for women from deprived areas, and uncertainty concerning the general provision of care, due to small sample size. It can therefore be argued that requiring informed consent for research based on patient records may act against the public interest in obtaining information concerning the success or failure of health policies to provide equitable care, according to need. Furthermore our main finding that the NHS provides equitable treatment for women with breast cancer would have been much less authoritative had it been based on smaller numbers.

If this work were considered to be audit rather than research the issues raised here would have been irrelevant. We viewed it to be research, as did several journals [10-12] because we were asking a new question of data collected for clinical purposes. There may be other examples where the boundary between audit and research is blurred sufficiently for research to be carried out in the name of audit, so avoiding this difficulty. This is confusing at best and unethical at worst.

## Conclusion

It is debatable whether such our original study would obtain ethical approval in the current climate, but if the NHS is to provide and monitor care for 100% of the population, it needs information from studies of this type. Although anonymisation of patient records should be carried out whenever possible, many research studies require re-identification of patients in a way that anonymisation would make impossible. We propose that research studies based on medical records, for the purpose of reviewing the coverage and equity of health care should, with appropriate safeguards, be recognised as a class of study for which individual patient consent is not required. With appropriate publicity, explanation, discussion and debate, we believe it should be possible to obtain public support for this approach.

## Competing interests

GCMW was a member of the Academy of Medical Sciences working group which produced the report "*Personal data for public good: using health information in medical research*."

## Authors' contributions

UM designed the study, carried out the data analysis and drafted the paper. GCMW designed the original study, and drafted the paper.

## Pre-publication history

The pre-publication history for this paper can be accessed here:


